# Integrating Inflammation-Responsive Prodrug with Electrospun Nanofibers for Anti-Inflammation Application

**DOI:** 10.3390/pharmaceutics14061273

**Published:** 2022-06-15

**Authors:** Jingjing Ye, Min Gong, Jian Song, Shu Chen, Qinghan Meng, Rui Shi, Liqun Zhang, Jiajia Xue

**Affiliations:** 1 State Key Laboratory of Organic-Inorganic Composites, Beijing University of Chemical Technology, Beijing 100029, China; 2017400077@mail.buct.edu.cn (J.Y.); gongmin@ustb.edu.cn (M.G.); 2021210312@buct.edu.cn (J.S.); 2017020580@mail.buct.edu.cn (S.C.); 2 Beijing Laboratory of Biomedical Materials, Beijing University of Chemical Technology, Beijing 100029, China; 3 College of Materials Science and Engineering, Beijing University of Chemical Technology, Beijing 100029, China; qhmeng@mail.buct.edu.cn; 4 Beijing Research Institute of Traumatology and Orthopaedics, Beijing Jishuitan Hospital, Beijing 100035, China

**Keywords:** enzyme-responsive drug release, anti-inflammatory, prodrug, electrospun nanofiber scaffold

## Abstract

Chronic inflammation plays a side effect on tissue regeneration, greatly inhibiting the repair or regeneration of tissues. Conventional local delivery of anti-inflammation drugs through physical encapsulation into carriers face the challenges of uncontrolled release. The construction of an inflammation-responsive prodrug to release anti-inflammation drugs depending on the occurrence of inflammation to regulate chronic inflammation is of high need. Here, we construct nanofiber-based scaffolds to regulate the inflammation response of chronic inflammation during tissue regeneration. An inflammation-sensitive prodrug is synthesized by free radical polymerization of the indomethacin-containing precursor, which is prepared by the esterification of *N*-(2-hydroxyethyl) acrylamide with the anti-inflammation drug indomethacin. Then, anti-inflammation scaffolds are constructed by loading the prodrug in poly(*ε*-caprolactone)/gelatin electrospun nanofibers. Cholesterol esterase, mimicking the inflammation environment, is adopted to catalyze the hydrolysis of the ester bonds, both in the prodrug and the nanofibers matrix, leading to the generation of indomethacin and the subsequent release to the surrounding. In contrast, only a minor amount of the drug is released from the scaffold, just based on the mechanism of hydrolysis in the absence of cholesterol esterase. Furthermore, the inflammation-responsive nanofiber scaffold can effectively inhibit the cytokines secreted from RAW264.7 macrophage cells induced by lipopolysaccharide in vitro studies, highlighting the great potential of these electrospun nanofiber scaffolds to be applied for regulating the chronic inflammation in tissue regeneration.

## 1. Introduction

Inflammation is a protective response in human-beings that prevents higher organisms from infection and injury [1]. The initial acute inflammatory response plays a vital role, acting as an indispensable phase in tissue healing and regeneration [2,3,4]. However, if not regulated tightly, acute inflammation may develop into chronic inflammation, which will cause an excessive release of noxious by-products and eventually lead to chronic diseases such as diabetic wounds [5,6], osteoarthritis [7,8], and others. Hence, it is important to ensure tissue regeneration by regulating chronic inflammation at the correct timing [2].

Clinically, oral administration of nonsteroidal anti-inflammatory drugs (NSAIDs) is often used to regulate inflammation, among which ibuprofen, indomethacin (IDCM), and diclofenac are commonly used [9,10]. However, with the existence of the carboxyl moieties in NSAIDs, long-term intake of NSAIDs can incur severe side effects [11,12], especially dose-dependent gastrointestinal disturbances [13]. For this purpose, it is especially urgent to alleviate the adverse effects of NSAIDs without limiting their anti-inflammatory activities. Local delivery of drugs to the injured tissue was intensively studied in recent years through physically loading drugs into various carriers for sustained release [14,15,16].

Additionally, anti-inflammation scaffolds have been constructed to regulate the inflammatory response during tissue regeneration. Among them, anti-inflammatory electrospun nanofibers play vital roles in the regeneration of skin [17,18], bone [19,20], blood vessel [21,22], and nerve [23,24] due to the nano-size architecture similar to the extracellular matrix [25] capable of delivering many types of drugs and bioactive materials [26]. For example, polyurethane/pluronic F127 nanofibers containing peppermint extract (an anti-inflammatory agent)-loaded-gelatin nanoparticles were prepared to promote diabetic wound healing [27]. Similarly, Lawsonia inermis was used as an anti-inflammatory agent and added to the gelatin-oxidized starch to generate nanofibers through electrospinning [28]. Additionally, the anti-inflammation drug ibuprofen was loaded into polylactic acid nanofibers to inhibit the proliferation of inflammatory macrophages [29].

Despite these progresses, most of the reported drug release systems were based on physical encapsulation to realize the release of the payloads to the tissues throughout the regeneration period, instead of in an inflammation-responsive manner. One promising solution to this problem represented by the prodrug strategies realized by grafting anti-inflammatory drugs onto the backbone of polymers [30] or peptides [31,32] through covalent bonding. Compared to normal tissues, inflammatory sites possess unique pathophysiological factors, such as reactive oxygen species [33,34], faintly acidic microenvironments [35], and higher levels of specific enzymes [36]. Among them, cholesterol esterase (CE), secreted by monocyte-derived macrophages, gradually accumulates to a distinctly high concentration around inflammatory sites as macrophages gather [37]. Particularly, CE has a remarkable capacity to cleave ester bonds selectively [38,39]. In this case, the targeted release of NSAIDs at the inflammatory sites can be achieved by covalently bonding NSAIDs onto polymer chains through ester bonds, utilizing the carboxylic acid moieties [39,40,41], which will greatly alleviate the adverse effects. For example, an anti-inflammatory polyprodrug was produced, grafting the anti-inflammatory drug IDCM by redox-responsive bonds to amphiphiles, achieving inflammation-triggered drug release characteristics [1]. Cui et al. produced an inflammation-sensitive prodrug with ester-linked anti-inflammatory drugs that could be cleaved by lipase, which is an important regulator during inflammation [40]. The selection of polymeric matrices needs further improvement to enhance therapeutic efficacy.

In view of the wide application of electrospun nanofibers in tissue regeneration, it’s feasible to use them as the matrix to load prodrugs. Especially, poly(ε-caprolactone) (PCL) is a decent candidate for tissue application by virtues of its biocompatible, degradable, and excellent electrospinning ability [42]. More importantly, the cleavage of ester bond in PCL can be significantly facilitated under the catalysis of CE, leading to the degradation of the polymer matrix and accelerated drug release. However, the deficiency in hydrophilicity and biologically active motifs restricts PCL alone from applying in tissue engineering [6,43]. Compared with a single material, composite nanofibrous scaffolds produced from natural and synthetic polymers provide better physicochemical properties, with the aim of satisfying the criteria of tissue regeneration [44]. Large numbers of studies employed PCL and gelatin as the matrix of electrospun nanofibers because gelatin can enhance cell adhesion and migration [45,46].

**Figure 1:**
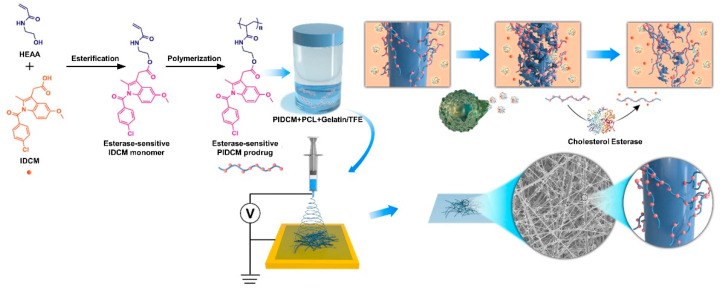
Schematic illustration of integrating inflammation-responsive prodrug with electrospun nanofibers for anti-inflammation application. The synthesis procedure of inflammation-responsive prodrug PIDCM, and schematic illustration of the enzyme-triggered release of IDCM from electrospun nanofibers loaded with inflammation-responsive prodrug.

Herein, we present an efficient platform for the on-demand release of anti-inflammatory drugs for chronic inflammation during tissue regeneration by integrating the inflammation-sensitive prodrug with the electrospun nanofiber scaffold, as shown in Figure 1. *N*-(2-hydroxyethyl) acrylamide (HEAA) was coupled with the anti-inflammation drug IDCM by mild esterification reaction for the monomer, which was subsequently free radical polymerized to obtain the inflammation-sensitive prodrug (PIDCM). Then, the anti-inflammation scaffolds were successfully constructed by loading the prodrug PIDCM into the PCL/gelatin electrospun nanofibers. CE, mimicking the inflammation environment, was adopted to catalyze the hydrolysis of the ester bonds both in the prodrug and the PCL/gelatin matrix, leading to the generation of IDCM and the subsequent release to the surrounding. The CE enzyme-triggered release profile of the anti-inflammatory drug IDCM from the scaffolds and the efficacy of the anti-inflammation scaffolds on relieving the inflammation in lipopolysaccharide (LPS)-induced RAW264.7 cells model at the cellular level were studied. The results demonstrate a significant difference in drug release, and much more IDCM could be triggered to release from the scaffolds with the help of the CE enzyme. Furthermore, upon incubation of the scaffolds with LPS-induced RAW264.7 cells, the scaffolds could effectively inhibit the cytokines secreted from the RAW264.7 cells and regulate the inflammatory responses. This work offers a facile and widely-applicable strategy for the fabrication of smart biomaterials with the stimuli-responsive capability to promote tissue regeneration by regulating chronic inflammation.

## 2. Materials and Methods

### 2.1. Materials

IDCM (99%) and *N, N*-Dimethylformamide (DMF, anhydrous, 99.8%) were purchased from Aladdin (Shanghai, China) and Alfa Aesar (Heysham, UK), respectively. Azobisisobutyronitrile (AIBN) and HEAA (98%) were purchased from TCI (Tokyo, Japan). *N*, *N’*-dicyclohexylcarbodiimide (DCC, 99%) and 4-dimethylaminopyridine (DMAP, 99%) were purchased from Acros Organics (Belgium). PCL (*M_n_* = 80 kDa) was obtained from Sigma-Aldrich (St. Louis, MI, USA). Gelatin (Type B) was obtained from Rousselot (Angoulême, France). Trifluoroethanol (TFE) was purchased from Aladdin (Shanghai, China). LPS (L2880-10MG) and CE (C9281-100UN) were purchased from Sigma-Aldrich (St. Louis, MI, USA). Cell counting kit-8 (CCK-8) was purchased from WISSEN. Alpha minimum essential medium (α-MEM), Dulbecco’s modified eagle medium (DMEM), phosphate buffer saline (PBS, pH = 7.4), and fetal bovine serum (FBS) were purchased from Gibco (New York, NY, USA). MC3T3-E1 mouse pre-osteoblasts cell, L929 fibroblast cell, and RAW264.7 mouse macrophages cell were purchased from Peking Union Medical College Hospital. 4′,6-diamidino-2-phenylindole (DAPI) was purchased from Solarbio (Beijing, China). Alexa Fluor 568-phalloidin was purchased from Invitrogen (California, USA). Interleukin (IL-6) ELISA kit was purchased from Neobioscience (Shenzheng, China). Nitrite (NO) detection kit was purchased from Nanjing Jiancheng Biology Engineering Institute (Nanjing, China).

### 2.2. Fabrication of Prodrug Loaded Electrospun Nanofibers

#### 2.2.1. Preparation of IDCM Monomer

The IDCM monomer (labeled as MIDCM) was synthesized by the esterification of hydroxyl groups of HEAA with carboxyl groups of IDCM. Typically, 2.15 g of IDCM (6 mmol), 2.07 g of HEAA (18 mmol), and 0.37 g of DMAP (3 mmol) were dissolved in anhydrous DMF (30 mL) under stirring, followed by moving to an ice-water bath. Then 3.71 g of DCC (18 mmol) was added as a dehydration catalyst. After stirring for 15 min, the temperature of the water bath was set at 40 °C. After 24 h, the reaction was stopped and the crude product was obtained through precipitating in deionized (DI) water, followed by purification using chromatography, leading to the final prodrug. The specific experimental operation was carried out according to the previous report [39].

#### 2.2.2. Preparation of IDCM Prodrug

MIDCM was polymerized by radical polymerization to prepare poly (MIDCM) (further labeled as PIDCM) [39]. The molecular weight of PIDCM was controlled by varying the reaction time. Briefly, 0.5 g of MIDCM (1.1 mmol) and 5 mg of AIBN (0.03 mmol) were firstly dissolved in 5 mL of DMF under a nitrogen atmosphere and then sealed. After stirring for 8 h and 36 h in an oil bath at 70 °C, respectively, the reactions were stopped, and the solutions were precipitated in DI water. After drying under vacuum for 24 h, PIDCM with two different molecular weights were obtained, and the *M*_n_ were measured by gel permeation chromatography (GPC, Waters 1525–2414 system, Wasters Corporation, Milford, MA, USA). Polystyrene and tetrahydrofuran were the standard reference and the eluent for calibrating the GPC, respectively. The columns were Waters Styragel HT3 THF, Waters Styragel HT4 THF, and Waters Styragel HT5 THF. The detector was the 2414 differential refractive index detector. The prodrugs with a molecular weight of about 3500 g/mol and 1400 g/mol were prepared and labeled as PIDCM_35_ and PIDCM_14_, respectively.

#### 2.2.3. Fabrication of Electrospun Nanofibers

PCL and gelatin were used as the matrix to fabricate the PIDCM prodrug-loaded electrospun nanofibers. Electrospinning was carried out according to the previous report [45,46], and the type of spinning machine was purchased from Beijing Xinrui Baina Technology Co., Ltd. (TEADFS-103, Beijing, China). In brief, PCL and gelatin (50/50 *w*/*w*) were dissolved in TFE to prepare the homogeneous electrospinning solution, and then the prodrugs PIDCM_35_ and PIDCM_14_ in the range of 0–60 wt.% were separately added to the electrospinning solutions. The solution was added to a 10 mL syringe and fed by a syringe pump at a rate of 1 mL/h. At the same time, a roller wrapped with aluminum foil was applied as the collector, and the rotating rate was set to 270 rpm. Between the needle and the grounded collector, a high voltage (12 KV) was applied. The distance between the needle and the ground collector was set as 15 cm. As shown in Table 1, the prepared electrospun nanofibers with PIDCM_35_ of 20 and 60 wt.% were labeled as PGPI_35_20 and PGPI_35_60, respectively. The electrospun nanofibers with PIDCM_14_ of 20, 40, and 60 wt.% were labeled as PGPI_14_20, PGPI_14_40, and PGPI_14_60, respectively. As a control, PCL/gelatin electrospun nanofiber was also prepared, which was labeled as PG0.

### 2.3. Morphology Characterization of Electrospun Nanofibers

The morphology of the electrospun nanofibers was characterized by scanning electron microscopy (SEM, S4800, Hitachi, Japan). The diameter of electrospun nanofibers on the SEM micrographs was measured by Image J software according to the previous report [47].

### 2.4. Chemical Characterization of Electrospun Nanofibers

The surface characterization of electrospun nanofibers was investigated by energy dispersive spectrometry (EDS) and X-ray photoelectron spectroscopy (XPS), which could provide the information of different elements on the surface. The Cl content of the electrospun nanofibers’ surfaces was investigated by EDS, which was carried out on a HORIBA X-Max20 detector (HORIBA Corporation, Kyoto, Japan) attached to the SEM. At the same time, the surface composition and functional groups of the electrospun nanofibers were investigated by XPS, which was performed on an ESCALAB 250 (Thermo Electron Corporation, Waltham, MA, USA) according to the previous report [48,49].

### 2.5. Hydrophilicity Characterization of Electrospun Nanofibers

The water contact angles of the electrospun nanofiber scaffolds were determined by a SL200A type Contact Angle Analyzer (SOLON TECH., Shanghai, China) at room temperature according to the previous report [45].

### 2.6. Cytotoxicity and Proliferation of Cells on the Surfaces

MC3T3-E1 and L929 cells are typical cells for testing cytotoxicity [50,51]. The viability of the cells proliferated on the electrospun nanofiber scaffolds loaded with PIDCM_35_ and PIDCM_14_ were evaluated using MC3T3-E1 osteoblasts and L929 fibroblasts by using CCK-8 assay, respectively. Briefly, the electrospun nanofiber scaffolds were cut into 13 mm diameter sheets with a 13 mm diameter cutter, sterilized by an Ultraviolet lamp, then fixed in a 24-well plate, and 4.0 × 10^3^ cells were plated onto the surfaces of samples carefully, followed by moving to an incubator at 37 °C. The number of cells that proliferated on the scaffolds at a determined time interval (day 1, 3, 5, and 7) was quantified using the CCK-8 assay referring to previous reports [39,52]. As for the electrospun nanofiber scaffolds loaded with PIDCM_35_, the samples co-cultured with cells were firstly washed with PBS three times, followed by fixing cell morphology via 3% glutaraldehyde. After dehydration and lyophilization, the morphology of MC3T3-E1 cells on the scaffolds loaded with PIDCM_35_ was observed by SEM. As for the electrospun nanofiber scaffolds loaded with PIDCM_14_, the samples co-cultured with cells were washed with PBS three times, and then L929 cells were stained by DAPI and Alexa Fluor 568-phalloidin and observed under a fluorescent inverted microscope (Axio Observer 3).

### 2.7. Drug Release Profiles

The drug release profiles of the PGPI_35_20 and PGPI_35_60 were determined as follows. Each sample was cut into 13 mm round discs, and all the samples were accurately weighed. Then, the samples were incubated at 37 °C in 1 mL PBS (pH 7.4) with or without CE (10 U/mL) to investigate the release kinetics of IDCM. High-performance liquid chromatography (HPLC) was used to detect the amount of released IDCM [39]. The percentage of IDCM release from triplicate samples was then determined based on the amount of IDCM in the prodrug incorporated into electrospun nanofiber scaffolds.

### 2.8. In Vitro Anti-Inflammatory Activity

RAW264.7 cells were used to evaluate the anti-inflammatory effect of the electrospun nanofiber scaffolds. Briefly, RAW264.7 cell suspension (5 × 10^5^ cells/mL, 1 mL/well) was added to a 24-well plate. After incubation for 24 h, LPS was added to the DMEM solution with 10% FBS at a concentration of 5 μg/mL, followed by replacing the old culture medium. After incubation for another 24 h, the LPS-treated RAW264.7 cells were incubated with PG0, PGPI_35_20, and PGPI_35_60 for 24 h and 48 h, respectively. Finally, the levels of IL-6 and NO in the supernatants were determined by the ELISA kit and Griess reaction, respectively [39].

### 2.9. Statistical Analysis

All quantitative data were expressed as mean ± standard deviation. OriginPro 8 software (Hampton, OriginLab) was used to perform the statistical analyses. The statistical differences between independent samples were performed by using the student’s *t*-test. * *p* < 0.05, ** *p* < 0.01, and *** *p* < 0.005 were regarded as statistically significant among independent groups.

## 3. Results and Discussion

### 3.1. Characterization of Prodrug

**Figure 2:**
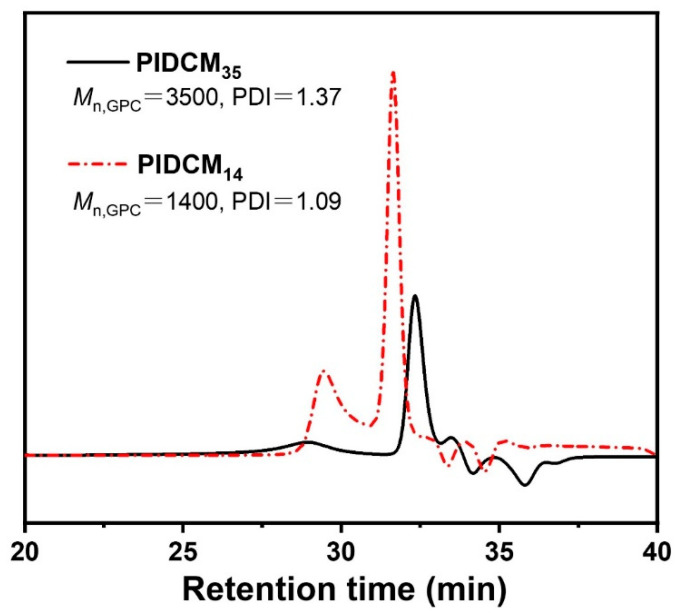
GPC curves of the prodrug with different molecular weights PIDCM_35_ and PIDCM_14_, respectively.

To investigate the influence of the different molecular weights of prodrugs on the anti-inflammatory properties of the electrospun nanofiber scaffolds: firstly, two PIDCM prodrugs with the molecular weight of 1400 g/mol and 3500 g/mol were readily obtained via free radical polymerization, respectively. The molecular weights of the two PIDCM prodrugs were measured using GPC, as shown in Figure 2, indicating the low polydispersity index. Then, the as-synthesized prodrug was incorporated with PCL/gelatin matrix to prepare the PIDCM-loaded electrospun nanofibers through electrospinning.

### 3.2. Characterization of Electrospun Nanofibers Loaded with the Prodrug

#### 3.2.1. Morphology of the Electrospun Nanofibers

**Figure 3:**
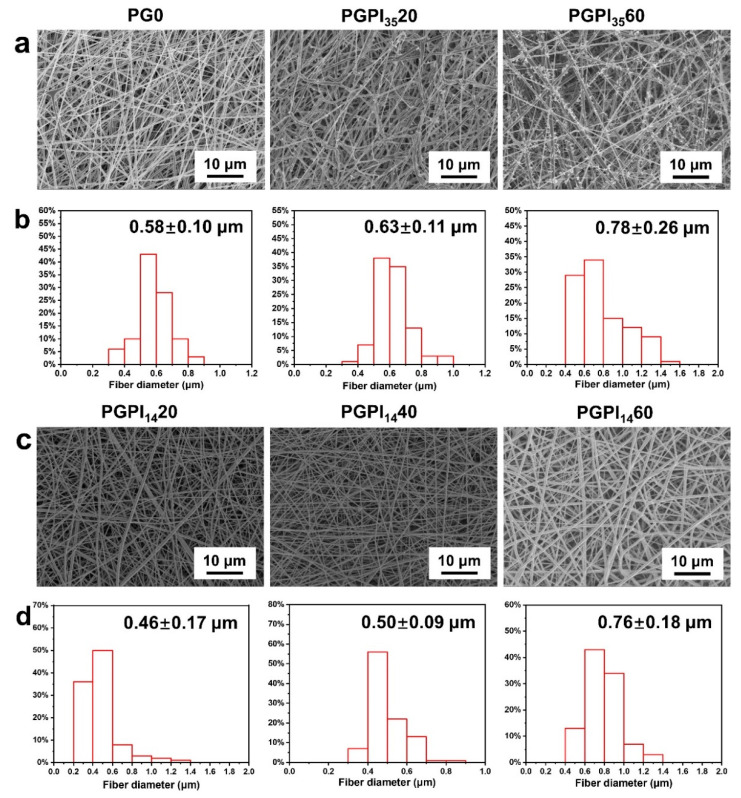
(**a**) SEM micrographs and (**b**) fiber diameter distribution of PG0, PGPI_35_20, and PGPI_35_60, respectively. (**c**) SEM micrographs and (**d**) fiber diameter distribution of PGPI_14_20, PGPI_14_40, and PGPI_14_60, respectively.

As shown in Figure 3a, SEM micrographs show that the PG0 nanofibers were randomly arranged with smooth surfaces, whereas the surfaces of PGPI_35_20 and PGPI_35_60 nanofibers contained needle-like bumps, and the number of the bumps increased with the increase in the content of PIDCM_35_. The average fiber diameters of PG0, PGPI_35_20, and PGPI_35_60 were 0.58 ± 0.10, 0.63 ± 0.11, and 0.78 ± 0.26 μm, respectively, as shown in Figure 3b. We also tested the fiber diameter distribution of PGPI_35_20 and PGPI_35_60 including the needle-like bumps in Figure S1, the average fiber diameters of PGPI_35_20 and PGPI_35_60 including the needle-like bumps were 0.72 ± 0.11 and 1.06 ± 0.27 μm, respectively. On the contrary, SEM micrographs (Figure 3c) of the PGPI_14_20, PGPI_14_40, and PGPI_14_60 nanofibers showed smooth topography and no apparent drug crystals. As shown in Figure 3d, the average diameters of PGPI_14_20, PGPI_14_40, and PGPI_14_60 were 0.46 ± 0.17, 0.50 ± 0.09, and 0.76 ± 0.18 μm, respectively, and the diameters of the nanofibers were increased with the increase in PIDCM_14_ content. Due to the higher molecular weight of PIDCM_35_ than PIDCM_14_, it was easier for PIDCM_35_ to precipitate from the nanofibers during electrospinning.

#### 3.2.2. Chemical Properties of the Electrospun Nanofibers

**Figure 4:**
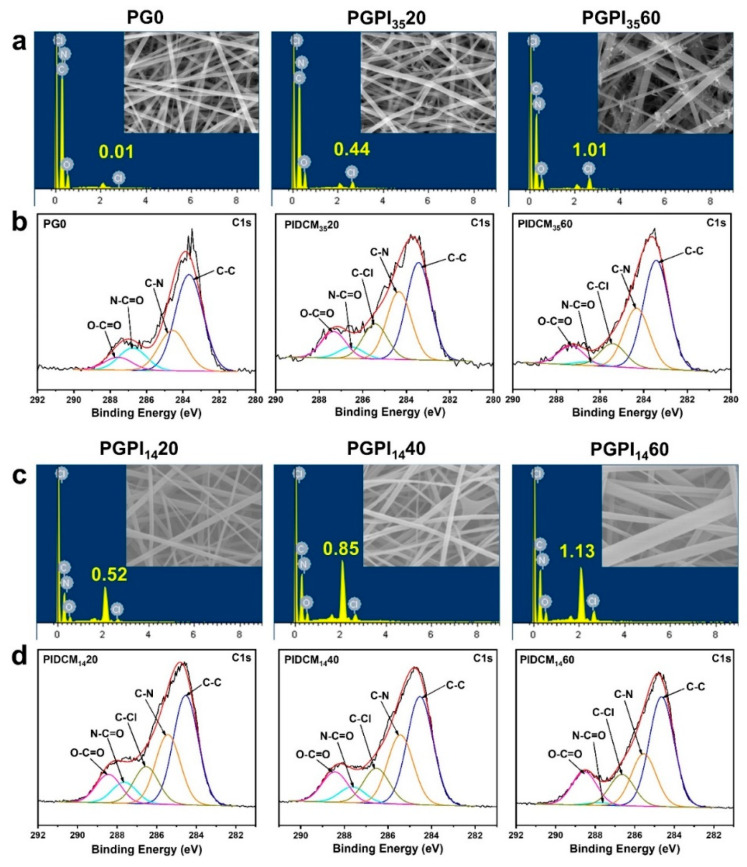
(**a**) The EDS spectra and (**b**) normalized C 1s XPS spectra of PG0, PGPI_35_20, and PGPI_35_60, respectively. (**c**) The EDS spectra and (**d**) normalized C 1s XPS spectra of PGPI_14_20, PGPI_14_40, and PGPI_14_60, respectively.

As shown in Figure 4a,c, the presence of Cl in PGPI_35_20, PGPI_35_60, PGPI_14_20, PGPI_14_40, and PGPI_14_60 were revealed by EDS, and their atomic percentages were 0.44, 1.01, 0.52, 0.85, and 1.13 at.%, respectively. As for the Cl in PG0, the atomic percentage was only 0.01 at.%, which was attributed to impurities or test deviation. The presence of Cl elements in PGPI_35_20, PGPI_35_60, PGPI_14_20, PGPI_14_40, and PGPI_14_60 electrospun nanofibers further indicated that PIDCM was dispersed within the PCL/gelatin matrix completely at the molecular level.

To further confirm the successful preparation of the PIDCM loaded into electrospun nanofibers, the surface composition and functional groups on electrospun nanofibers were investigated by XPS. Figure 4b,d show the C 1s spectra of the electrospun nanofibers. In the C 1s spectrum, the surface of pure PG0 nanofibrous mats presented four expected peaks with binding energy at 283.7, 284.6, 286.8, and 287.6, indicating the existence of four carbon regions of C–C, C–N, N–C=O, and O–C=O, respectively. After loading with PIDCM_35_ and PIDCM_14_, the chemical compositions of the nanofiber surfaces were significantly changed. For the other five samples, one new C 1s peak with the binding energy of about 285.4, 285.4, 286.55, 286.55, and 286.65 eV appeared, which could be attributed to the new carbon region of C–Cl for PGPI_35_20, PGPI_35_60, PGPI_14_20, PGPI_14_40, and PGPI_14_60, respectively. These results suggested that PIDCM_35_ and PIDCM_14_ were loaded in the PCL-gelatin nanofibers.

#### 3.2.3. Effects of PIDCM Encapsulation on Hydrophilicity of Scaffolds

**Figure 5:**
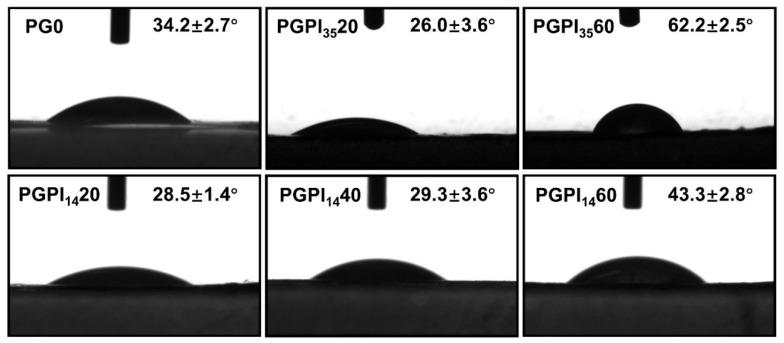
The water contact angles of PG0, PGPI_35_20, PGPI_35_60, PGPI_14_20, PGPI_14_40, and PGPI_14_60, respectively.

Hydrophobicity of materials plays a vital role in tissue regeneration [45]. The hydrophilicity of the scaffolds was investigated by the water contact angles test, as shown in Figure 5. Among them, the water contact angles of the electrospun nanofiber scaffolds loaded with PIDCM_35_ or PIDCM_14_ were increased with the increase in PIDCM content. Specifically, PGPI_35_20 and PGPI_14_20 had good hydrophilicity, and their water contact angles were 26.0° and 28.5°, respectively, smaller than the contact angle of PG0 without drug loading. The hydrophilicity property of the scaffolds could provide them with a stronger protein adsorption capability, which plays an important role in providing cues to interact with cells and surrounding tissues.

### 3.3. In Vitro Biocompatibility of Electrospun Nanofibers Loaded with Prodrug

**Figure 6:**
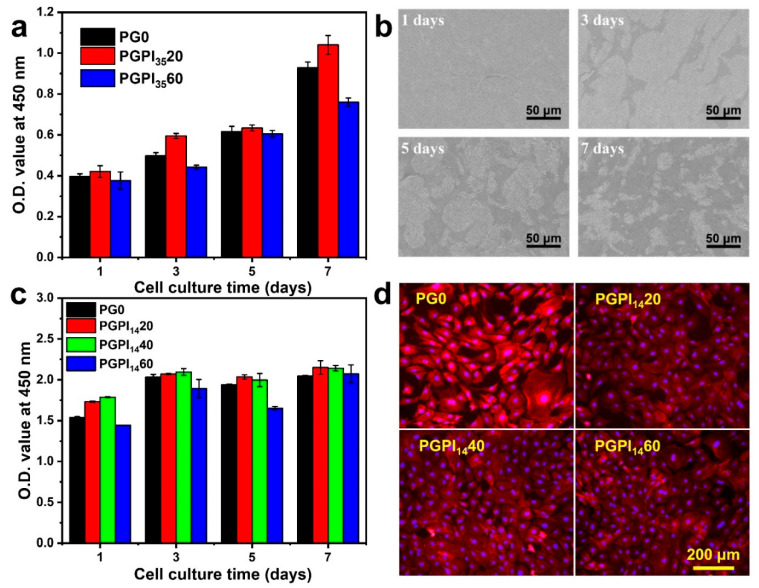
(**a**) O.D. values of MC3T3-E1 cells adhered to PG0, PGPI_35_20, and PGPI_35_60 for 1, 3, 5, and 7 days, respectively. (**b**) SEM micrographs of MC3T3-E1 cells proliferated on PGPI_35_20 for 1, 3, 5, and 7 days, respectively. (**c**) O.D. values of L929 cells adhered to PG0, PGPI_14_20, PGPI_14_40, and PGPI_14_60 for 1, 3, 5, and 7 days, respectively. (**d**) Fluorescence images of the L929 cells cultured on PG0, PGPI_14_20, PGPI_14_40, and PGPI_14_60 for 7 days, respectively. The F-actins of the cells were stained with Alexa Fluor 568-phalloidin (red), whereas cell nuclei were stained with DAPI (blue).

For cell-material interactions, attachment and proliferation are the first stages, thus the biocompatibility of material has a significant effect on the proliferation and morphology of cells [45]. Among them, MC3T3-E1 cells and L929 cells are widely used to test the biocompatibility of materials [45,46]. Thus, the cell proliferation assay of PG0, PGPI_35_20, and PGPI_35_60 was tested by MC3T3-E1 cells. Meanwhile, the cell proliferation assay of PGPI_14_20, PGPI_14_40, and PGPI_14_60 was tested by L929 cells. As shown in Figure 6a, the numbers of MC3T3-E1 cells progressively increased during the 7 days, indicating that MC3T3-E1 cells were adhered and in a proliferative state on PG0, PGPI_35_20, and PGPI_35_60. Similarly, PG0, PGPI_35_20, and PGPI_35_60 had good effects on promoting cell proliferation and showed good biocompatibility. At the same time, the morphologies of the MC3T3-E1 cells proliferating on PGPI_35_20 for 1, 3, 5, and 7 days were observed by SEM (Figure 6b), respectively. The expanding area of MC3T3-E1 cells on the surface of the electrospun nanofibers increased with the extension of the co-culture time, and the cells on the surface of the PGPI_35_20 reached 70–90% confluence on day 7.

As shown in Figure 6c, the numbers of L929 cells on PG0, PGPI_14_20, PGPI_14_40, and PGPI_14_60 were continuously increased during 7 days of incubation, indicating the scaffolds loaded with prodrugs were nontoxic and supported cell proliferation. As shown in the fluorescence microscopy images (Figure 6d), the cells reached 70–90% confluence after incubation for 7 days. Furthermore, L929 cells exhibited healthy morphologies after incubation with all groups. We can conclude that the PGPI_14_20, PGPI_14_40, and PGPI_14_60 could support the proliferation of L929 cells.

### 3.4. In Vitro Drug Release Profile of Electrospun Nanofibers Loaded with Prodrug

The prodrug encapsulation efficiency is significantly influenced by the interaction between the PCL/gelatin polymer matrix chains and PIDCM prodrug molecules [45]. PIDCM is hydrophobic, contains carbonyl groups and amide bonds, and is capable of interacting with the hydroxyl and carboxyl groups of gelatin chains via hydrogen bonding. In the system, the cleavage of ester groups depended on the slow hydrolytic and fast enzymatic cleavage. Compared to PIDCM_35_, PIDCM_14_ was more likely to be released from the nanofibers through diffusion due to its smaller molecular weight, which means a less controllable release manner [40]. Our study aimed to design an inflammation-responsive scaffold, which could release anti-inflammatory drugs under the stimulation of the inflammation to regulate the excessive inflammation. Therefore, we chose PGPI_35_20 and PGPI_35_60 to study the release profile of IDCM from the electrospun nanofiber scaffolds upon enzyme stimulation.

**Figure 7:**
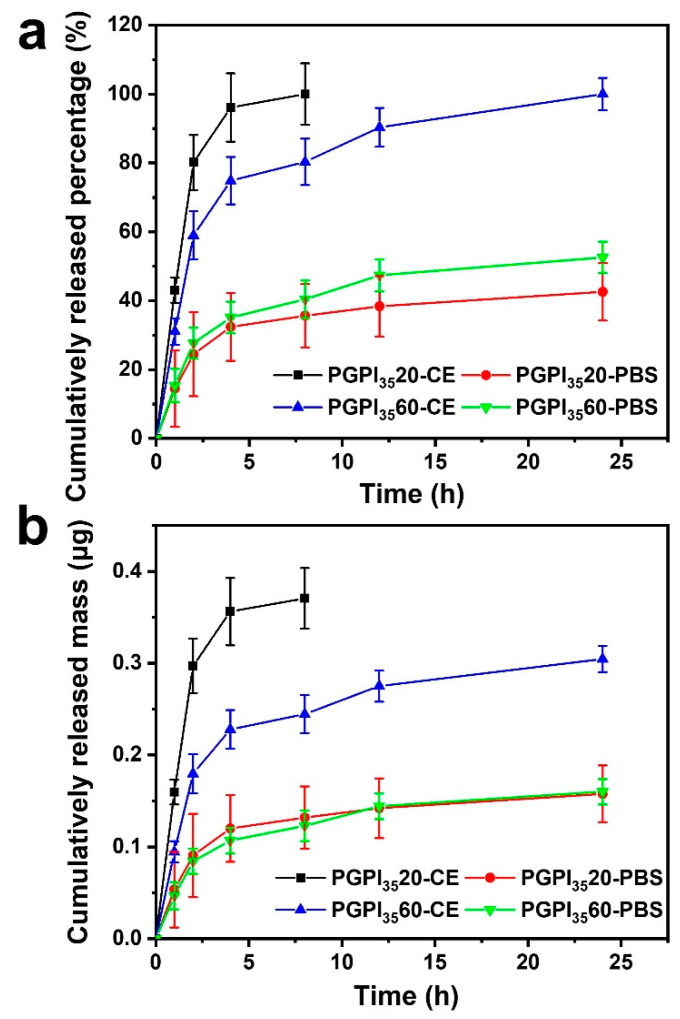
Cumulative released (**a**) percentage and (**b**) mass of IDCM from the electrospun nanofiber scaffolds loaded with different contents of PIDCM_35_ with or without CE (10 U/mL) in the PBS solution, respectively (*n* = 3).

Firstly, we tested the drugs after released from prodrugs by mass spectrum (Figure S2), in which the peak at 358.0845 was corresponding to IDCM. As shown in Figure 7, the enzyme-rich body fluid of living organisms during the occurrence of inflammation was mimicked by the CE enzyme. The cumulatively released percentage of IDCM at different time points was determined and calculated according to the standard curve [39]. For PGPI_35_20 and PGPI_35_60, the loaded amounts were 0.371 ± 0.033 μg and 0.305 ± 0.014 μg in one sample, respectively. In the absence of CE, both PGPI_35_20 and PGPI_35_60 showed an extremely slow release of IDCM and the total amounts of released IDCM were less than 50% in 24 h. In contrast, both PGPI_35_20 and PGPI_35_60 showed a significantly rapid release of IDCM in the presence of CE. Specifically, 100% IDCM was released from PGPI_35_20 in the first 8 h, and 100% IDCM was released from PGPI_35_60 in 24 h, showing the enzyme-triggered release behavior of the PGPI_35_20 and PGPI_35_60. Therefore, it can be concluded that the PGPI_35_20 and PGPI_35_60 had the capabilities of delivering the drug component IDCM under the stimulation of the CE enzyme, allowing for the smart and on-demand drug release to avoid the chronic inflammation that occurs during tissue regeneration.

### 3.5. In Vitro Evaluation of the Anti-Inflammatory Effect of Electrospun Nanofibers Loaded with Prodrug

**Figure 8:**
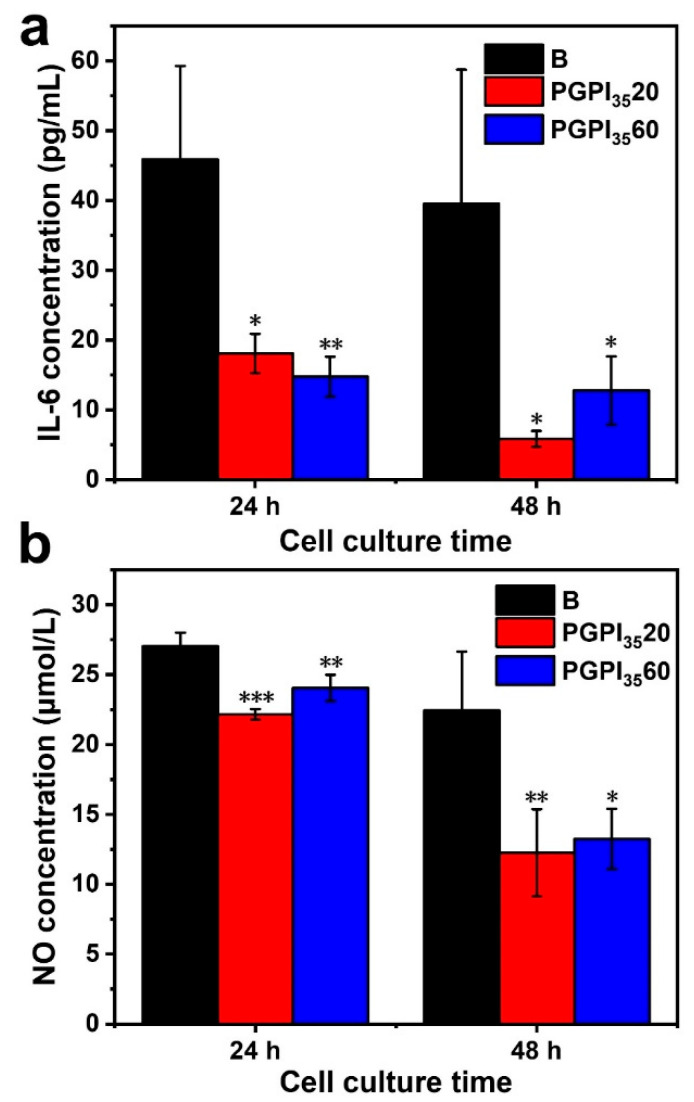
Effects of electrospun nanofiber scaffolds containing different content of PIDCM_35_ on the expressions of (**a**) IL-6 and (**b**) NO secreted from LPS-induced RAW264.7 cells, respectively. Cells were treated with electrospun nanofiber scaffolds in the presence of LPS (5 μg/mL) for 24 h (*n* = 3, * *p* < 0.05, ** *p* < 0.01, and *** *p* < 0.005).

RAW264.7 cells can secrete various kinds of cytokines under the stimulation of LPS [1,36], such as IL-6 and NO [53]. The anti-inflammatory effect of PGPI_35_20 and PGPI_35_60 were evaluated using the inflammation model according to the previous study [39]. As shown in Figure 8, compared with the blank group, the concentration of IL-6 and NO were remarkably decreased when the RAW264.7 cells were cultured with PGPI_35_20 and PGPI_35_60, indicating that they could successfully suppress the inflammation reaction. With the extension of the co-culture time of PGPI_35_20 and PGPI_35_60 with RAW264.7 cells, the levels of IL-6 (Figure 8a) and NO (Figure 8b) secreted from the LPS-induced cells were decreased, demonstrating that more content of IDCM was released from PGPI_35_20 and PGPI_35_60 through the inflammation-triggered release. There was no significant difference between PGPI_35_20 and PGPI_35_60, indicating the anti-inflammatory efficiency was similar between these two groups, due to the concentration of CE enzyme secreted from the RAW264.7 cells was in a certain level, which limits the amounts of ester linkages of PGPI_35_20 and PGPI_35_60 that could be cleaved by the CE. Taken together, the electrospun nanofiber scaffold loaded with the prodrug could inhibit the inflammation response, which will be beneficial for tissue regeneration.

## 4. Conclusions

In summary, enzyme-sensitive prodrugs were synthesized through the free radical polymerization of the IDCM-containing precursor and then loaded into PCL/gelatin electrospun nanofibers to develop an inflammation-responsive nanofiber scaffold. A large amount of IDCM was released from the anti-inflammation electrospun nanofiber scaffold under the stimulation of CE, whereas there was a minimal release of the drug in the absence of enzyme. Moreover, the inflammatory response could be significantly attenuated after incubating the scaffolds with LPS-treated RAW264.7 cells. CE was secreted by macrophages to a distinctly high concentration around inflammatory sites, further triggering the hydrolysis of ester bonds both in the prodrug and PCL/gelatin, resulting in the intensified degradation of the matrix and the rapid release of generated IDCM to relieve the chronic inflammatory response. This study offers a feasible and wide applicable strategy to deliver drugs in a smart, responsive, and effective manner and demonstrates great potential to be applied for regulating chronic inflammation during tissue regeneration. In addition to nanofiber scaffolds, the prodrug can also be incorporated into other types of scaffold matrix, such as 3D printed scaffolds and hydrogels, to suit different applications.

## Figures and Tables

**Table 1:** The abbreviations for PGPI_35_ and PGPI_14_.

Abbreviations	PIDCM_35_ (wt.%)	PIDCM_14_ (wt.%)
PGPI_35_20	20	0
PGPI_35_60	60	0
PGPI_14_20	0	20
PGPI_14_40	0	40
PGPI_14_60	0	60

## Data Availability

The data presented in this study are available on request from the corresponding author.

## Supplementary Materials

The following supporting information can be downloaded at: www.mdpi.com/article/10.3390/pharmaceutics14061273/s1, Figure S1. (a) SEM micrographs and (b) fiber diameter distribution of PGPI_35_20 and PGPI_35_60 including the needle-like bumps, respectively. Figure S2. The mass spectrum of the solution containing the released drug.
