# Supplementary material for: Integrating Inflammation-Responsive Prodrug with Electrospun Nanofibers for Anti-Inflammation Application
Source: Pharmaceutics. 2022 Jun 15;14(6):1273. doi: 10.3390/pharmaceutics14061273 (PMC9229020; doi:10.3390/pharmaceutics14061273)
Supplement: Supplementary file 1 [file pharmaceutics-14-01273-s001.zip › pharmaceutics-1738039-supplementary.pdf]

pharmaceutics

Integrating inflammation-responsive prodrug with electrospun nanofibers for anti-inflammation application

Jingjing Ye [1,2], Min Gong [1,2], Jian Song [1,2], Shu Chen [1,2], Qinghan Meng [3], Rui Shi [4,*], Liqun Zhang [1,2,*] and Jiajia Xue [1,2,*]

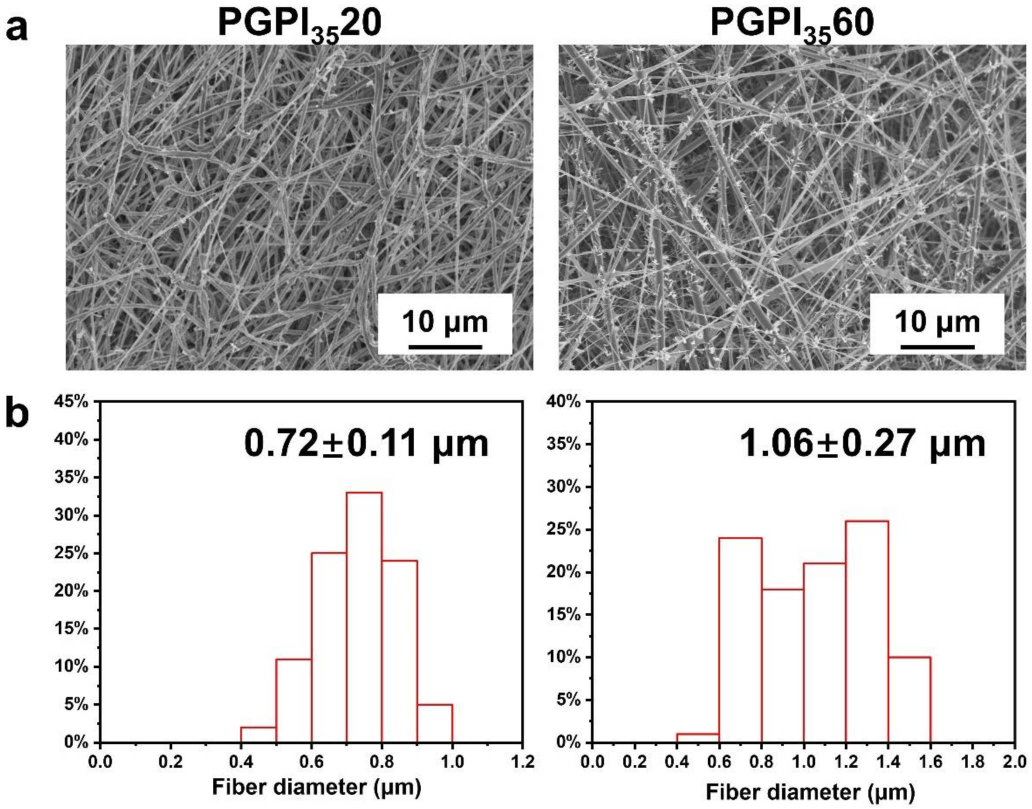

Figure S1. (a) SEM micrographs and (b) fiber diameter distribution of PGPI$_{35}$20 and PGPI$_{35}$60 including the needle-like bumps, respectively.

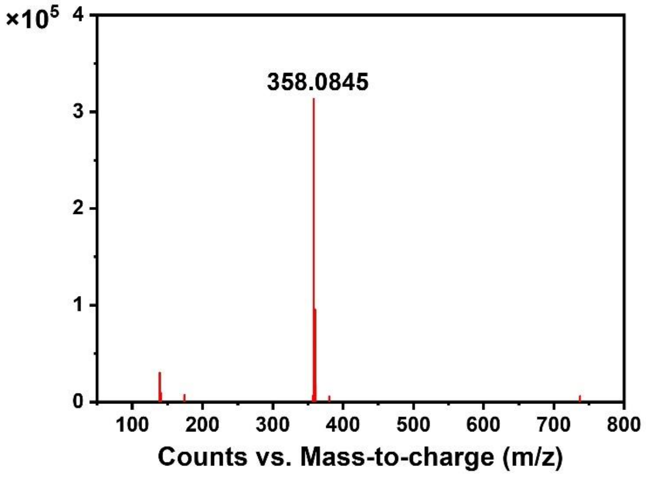

Figure S2. The mass spectrum of the solution containing the released drug.
